# *CYP3A5* Genotype as a Potential Pharmacodynamic Biomarker for Tacrolimus Therapy in Ulcerative Colitis in Japanese Patients

**DOI:** 10.3390/ijms21124347

**Published:** 2020-06-18

**Authors:** Yuki Yamamoto, Hiroshi Nakase, Minoru Matsuura, Shihoko Maruyama, Satohiro Masuda

**Affiliations:** 1 Department of Clinical Pharmacology and Therapeutics, Kyoto University Hospital, 54 Kawaharacho, Shogoin, Sakyo-ku, Kyoto 606-8507, Japan; ykto92@kuhp.kyoto-u.ac.jp (Y.Y.); smaruyam@kuhp.kyoto-u.ac.jp (S.M.); 2 Department of Clinical Pharmacology and Biopharmaceutics, Graduate School of Pharmaceutical Sciences, Kyushu University, 3-1-1 Maidashi, Higashi-ku, Fukuoka 812-8582, Japan; 3 Department of Gastroenterology and Hepatology, Sapporo Medical University School of Medicine, Minami 1-jo Nishi 17-chome, Chuo-ku, Sapporo, Hokkaido 060-8556, Japan; hiro_nakase@sapmed.ac.jp; 4 Department of Gastroenterology and Hepatology, Graduate School of Medicine, Kyoto University, 54 Kawaharacho, Shogoin, Sakyo-ku, Kyoto 606-8507, Japan; mmatsuura@ks.kyorin-u.ac.jp; 5 Department of Gastroenterology and Hepatology, Kyorin University School of Medicine, 6-20-6, Shinkawa, Mitaka-city, Tokyo 181-8611, Japan; 6 Department of Pharmacy, International University of Health and Welfare Narita Hospital, 852 Hatakeda, Narita 286-0124, Japan

**Keywords:** mucosal concentration, clinical activity index, multidrug resistance 1, polymorphism, inflammatory bowel disease

## Abstract

Tacrolimus has been used to induce remission in patients with steroid-refractory ulcerative colitis. It poses a problem of large individual differences in dosage necessary to attain target blood concentration and, often, this leads to drug inefficacy. We examined the difference in mRNA expression levels of ATP binding cassette transporter B1 (ABCB1) between inflamed and non-inflamed tissues, and the influence of *CYP3A5* genotype on tacrolimus therapy. The mRNA expression of CYP3A4 in colonic mucosa and that of cytochrome p450 3A5 (CYP3A5) and ABCB1 in inflamed and non-inflamed areas were examined in 14 subjects. The mRNA expression levels of CYP3A5 were higher than that of CYP3A4. The mRNA expression of ABCB1 was lower in the inflamed than in the non-inflamed mucosa, despite that of CYP3A5 mRNA level being not significantly changed. Hence, the deterioration of the disease is related to the reduction of the barrier in the inflamed mucosa. The relationship between *CYP3A5* genotype and blood concentration, dose, and concentration/dose (C/D) ratio of tacrolimus in 15 subjects was studied. The tacrolimus dose to maintain equivalent blood concentrations was lower in CYP3A5*3/*3 than in CYP3A5*1 carriers, and the C/D ratio was significantly higher in the latter. Thus, *CYP3A5* polymorphism information played a role in determining the initial dose of tacrolimus. Furthermore, since the effect of tacrolimus appears earlier in CYP3A5*3/*3 than in CYP3A5*1/*1 and *1/*3, it seems necessary to change the evaluation time of therapeutic effect by *CYP3A5* genotype. Additionally, the relationship between *CYP3A5* genotype and C/D ratio of tacrolimus in colonic mucosa was investigated in 10 subjects. Tacrolimus concentration in the mucosa was two-fold higher in CYP3A5*3/*3 than in CYP3A5*1 carriers, although no significant difference in tacrolimus-blood levels was observed. Therefore, the local concentration of tacrolimus affected by *CYP3A5* polymorphism might be related to its therapeutic effect.

## 1. Introduction

Conventionally, 5-aminosalicylic acid (5-ASA or mesalazine), steroids, calcineurin inhibitors, etc., have been used to treat ulcerative colitis (UC) [1,2]. However, in recent years, numerous biological products have been developed, and treatment options for UC are expanding [3]. Tacrolimus, which was first used as an immunosuppressant following an organ transplantation, is now being used in the management of various autoimmune diseases in a number of patients [4,5]. Therefore, the appropriate knowledge of an existing drug, including its merits and demerits in comparison to new drugs, can help make better therapeutic choices.

Tacrolimus is used to induce remission in patients with steroid-refractory UC. The drug binds to the FK506-binding protein 12 (FKBP12) and inhibits calcineurin. As a result, the activation of nuclear factor of activated T-cells (NFAT), a substrate of calcineurin, is inhibited. The NFAT-induced transcription of interleukin 2 and interferon γ in the nucleus is also inhibited. These cytokines promote the proliferation of helper T-cells; tacrolimus suppresses T-cell proliferation and thus exerts its immunosuppressive activity [6,7].

Tacrolimus is effective in UC at a target trough blood concentration of 10–15 ng/mL [4,8]. However, the side effects of tacrolimus including nephropathy, headache, and tremors related to the trough blood levels deter its use [9,10,11]. Furthermore, since individual differences in the pharmacokinetics of tacrolimus are large, it is difficult to adjust the dosage [12,13].

It has been reported that individual differences in pharmacokinetics and efficacy are due to the varying expression levels and activities of metabolic enzymes and transporters, which depend on genetic polymorphism [14].

Tacrolimus is metabolized by cytochrome p450 3A4 (CYP3A4) and CYP3A5 in the gastrointestinal mucosa and liver, and then excreted into the gastrointestinal lumen and bile by P-glycoprotein (Pgp) encoded by the ATP-binding cassette transporter B1 (ABCB1, other name: Multidrug resistance 1 (MDR1)) gene [15,16,17]. It is reported that the influence of CYP3A5 is greater than that of CYP3A4 on the metabolism of tacrolimus [18,19,20]. However, in the *CYP3A5* genotype, the efficacy of tacrolimus is affected and, therefore, a dose adjustment based on this genotype is required after organ transplantation [21,22,23]. Therefore, in the treatment of UC, the pharmacokinetics and effects of tacrolimus may be altered in the *CYP3A5* genotype [24]. We examined the relationship between the *CYP3A5* genotype (*1 allele and *3 allele (s776746)) and clinical activity index (CAI), a disease activity index in UC, including the measurement of tissue concentration of tacrolimus in colon mucosa.

P-glycoprotein, a gene product of ABCB1 (or other name is MDR1) is expressed in several organs including the liver and kidney and plays a role in pumping out drugs and exogenous compounds from the cells [25]. Therefore, a decrease in Pgp is considered as to be not only responsible for altering the pharmacokinetics of tacrolimus, but is also attributed to the worsening of the disease condition of UC [26,27]. Based on these backgrounds, the relationship between the degree of inflammation and ABCB1 mRNA-expression level was used as a positive control for the sampling of biopsy specimens for examining the mRNA expression levels of CYP3A4 and CYP3A5 in mucosal tissues.

## 2. Results

### 2.1. The mRNA Expression of CYP3A4, CYP3A5, and ABCB1

**Figure 1:**
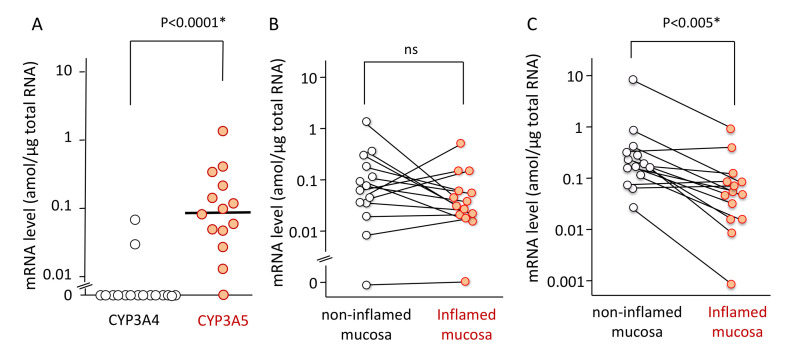
The mRNA expression levels of CYP3A4 and CYP3A5 in colonic or rectal mucosa (**A**), the mRNA expression levels of CYP3A5 in non-inflamed and inflamed mucosa (**B**), and the mRNA expression levels of ABCB1 in non-inflamed and inflamed mucosa (**C**). (**A**) Among 14 mucosal biopsy specimens, the mRNA expression level of CYP3A4 and CYP3A5 in 12 specimens and 1 specimen, respectively, were below the detection limit (0.01 amol/µg total RNA). The bar shows the median for all mRNA expression levels including data below the detection limitation whose value was set as zero. Statistical analysis was performed using the Mann–Whitney’s *U*-test. (**B**) The mRNA expression level of CYP3A5 in inflamed colorectal mucosal was compared to non-inflamed mucosa in 14 UC patients. Among them, the measurement was below the detection limit (0.01 amol/µg total RNA) in a specimen. Each line represents the non-inflamed and inflamed sample derived from the same patient. (**C**) The mRNA expression level of ABCB1 in inflamed colorectal mucosal was compared to non-inflamed mucosa in 14 UC patients. Each line represents the non-inflamed and inflamed sample derived from the same patient. Statistical analysis was performed using the paired *t*-test with logarithmical values.

Patient characteristics including age, sex, and extent of UC are listed in Table 1. The mRNA expression level of CYP3A5 was higher than that of CYP3A4 in colonic or rectal mucosa. The expression levels of CYP3A4 ranged from <0.01 (not detected) to 0.07 amol/µg total RNA (median < 0.01 amol/µg), while these values for CYP3A5 ranged from < 0.01 to 1.37 amol/µg total RNA (median 0.09 amol/µg). There was significant difference (*p* < 0.0001) (Figure 1A).

The mRNA expression of CYP3A5 was not significantly changed between non-inflamed and inflamed mucosa (Figure 1B). In contrast, the mRNA expression of ABCB1 was reduced in the inflamed regions as opposed to the non-inflamed regions in patients (median 0.23 [0.03–9.62], 0.07 [0.001–1.08] amol/µg, respectively). There was a significant difference (*p* < 0.005) (Figure 1C). Because the mRNA expression levels of CYP3A4 was almost negative in the non-inflamed biopsy specimens, those in the inflamed mucosa were not examined.

### 2.2. Blood Concentration, Dose, and Concentration/Dose (C/D) Ratio of Tacrolimus

Patient characteristics based on *CYP3A5* genotypes reflecting CYP3A5 protein expression are summarized in Table 2. We examined the influence of *CYP3A5* genotype on tacrolimus therapy comparing two groups between CYP3A5 expressor (*CYP3A5* *1/*3) and nonfunctional (*CYP3A5* *3/*3). The chi-squared test was used to verify that the *CYP3A5* genotype frequency distribution for the present patients was consistent with the Hardy–Weinberg equilibrium (*p* > 0.05).

**Figure 2:**
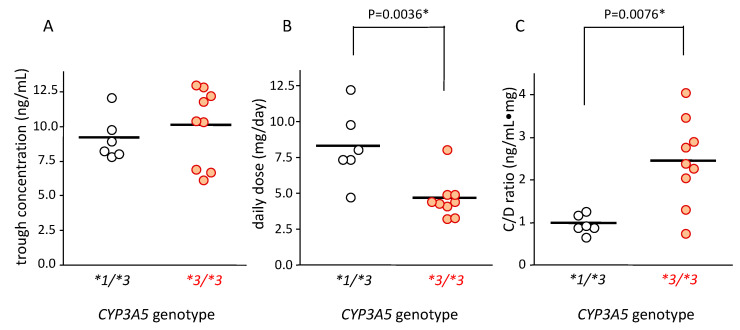
Relationship between *CYP3A5* genotype and blood concentration (**A**), dose (**B**), and concentration/dose (C/D) ratio (**C**) of tacrolimus. The bar shows the mean in each group (**A**,**B**) and the median for the C/D ratio of tacrolimus. (**A**) The average concentration of tacrolimus trough level in each patient was plotted. The mean trough concentration in patients carrying CYP3A5*1/*3 (n = 6) was similar to that of patients carrying CYP3A5*3/*3 (n = 9). (**B**) The average dose of tacrolimus in each patient was plotted. The mean value of average dose of tacrolimus in patients carrying CYP3A5*1/*3 was higher than that with CYP3A5*3/*3 (*p* = 0.0036 using Student’s *t*-test). (**C**) The average concentration/dose ratio of tacrolimus was plotted for each patient. Each point represents an average of 10 points. Bar shows the median value of all plotted points. Statistical analysis was performed using the Mann–Whitney’s *U*-test.

The tacrolimus concentration in blood in patients with CYP3A5*1/*3 and CYP3A5*3/*3 was 9.22 ± 1.61 ng/mL (mean ± standard deviation (SD)) and 10.12 ± 2.77 ng/mL (mean ± SD), respectively; there was no significant difference between these groups (Figure 2A).

The tacrolimus dose in patients carrying the CYP3A5*1/*3 genotype (8.31 ± 2.55 mg/day [mean ± SD]) was 1.7-fold higher than that in patients carrying CYP3A5*3/*3 (4.69 ± 1.42 mg/day (mean ± SD)) (*p* = 0.0036) (Figure 2B).

The median value of blood C/D ratio of tacrolimus in patients with the CYP3A5*1/*3 and CYP3A5*3/*3 genotypes was 0.93 (0.68–1.28) and 2.40 (0.78–4.07), respectively. This value was approximately 2.5 times higher in patients with the CYP3A5*3/*3 than in those with the CYP3A5*1/*3 genotype, which was significantly different (*p* = 0.0076) (Figure 2C).

### 2.3. Association between the Clinical Outcome and CYP3A5 Genotype in Tacrolimus Therapy

Clinical information including the clinical activity index (CAI) values were obtained on days 0, 14, and 30 after the start of tacrolimus therapy. The relationship between the ratio of variation in CAI values between days 0 and 14, and days 14 and 30, and the *CYP3A5* genotype was examined. Furthermore, in order to confirm the effectiveness of tacrolimus, the presence or absence of therapy with biologics, anti-tumor necrosis factor (TNF) agents, and surgical intervention for 30 months after the start of tacrolimus therapy was investigated.

**Figure 3:**
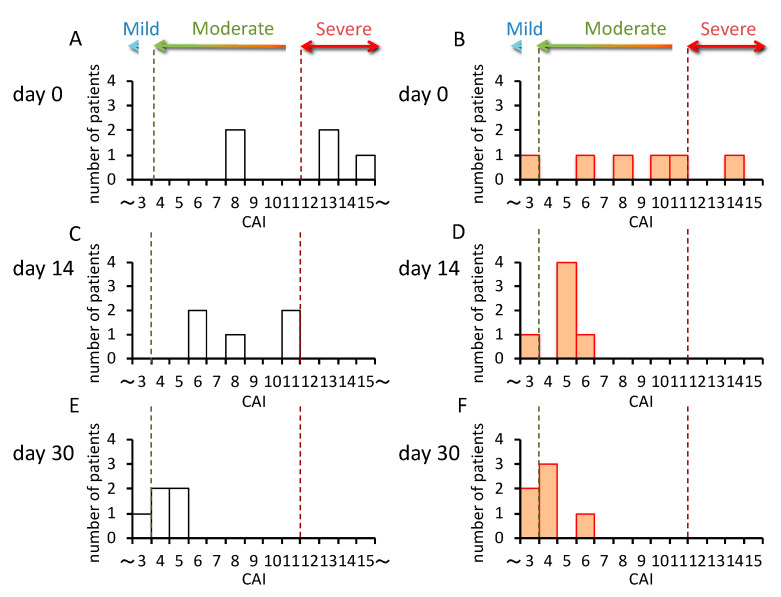
Change in the clinical activity index (CAI) in the *CYP3A5* genotype after the start of tacrolimus therapy. The white bars indicate the number of patients carrying the CYP3A5*1/*3 genotypes (CYP3A5 expressor, n = 5) (**A**,**C**,**E**). The orange bars indicate patients carrying the CYP3A5*3/*3 genotype (CYP3A5 nonfunctional, n = 6) (**B**,**D**,**F**).

**Figure 4:**
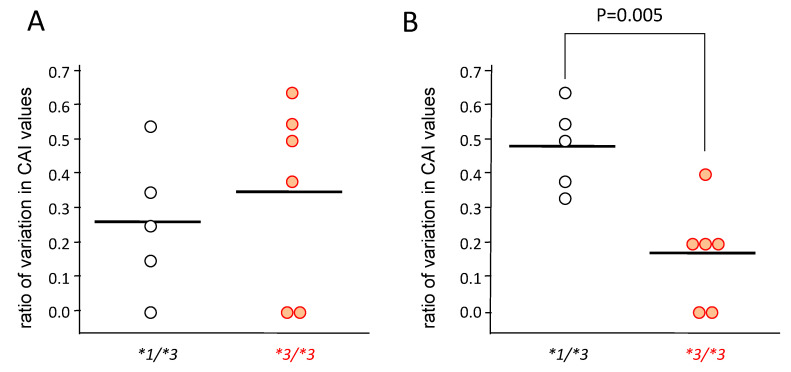
The rate of change in clinical activity index (CAI) in the *CYP3A5* genotype after the start of tacrolimus therapy from day 0 to day 14 (**A**) and from day 14 to day 30 (**B**). The value of vertical axis was obtained by dividing the value of difference between the CAI value at day 14 from it at day 0 by the CAI value at day 0 ((CAI at day 0–CAI at day 14)/CAI at day 0) (**A**), and by dividing the value of difference between the CAI value at day 30 from it at day 14 by that at day 14 ((CAI at day 14–CAI at day 30)/CAI at day 14) (**B**). Statistical analysis was performed using the Student’s *t*-test.

In patients carrying CYP3A5*1/*3 (n = 5), the CAI values decreased gradually from day 0 to day 30 (Figure 3A,C,E), but in patients carrying CYP3A5*3/*3 (n = 6), the CAI values rapidly decreased from day 0–14, and did not change much from day 14–30 (Figure 3B,D,F). Furthermore, although the rate of change was large in CYP3A5*3/*3 from day 0–14, significantly higher rates of change were seen in patients carrying CYP3A5*1/*3 from day 14–30 (*p* = 0.005) (Figure 4).

### 2.4. Blood and Mucosal Concentrations and the Mucosal Concentration/Dose of Tacrolimus

We considered the possibility that pharmacological efficacy of tacrolimus was affected by its mucosal concentration dependent on the *CYP3A5* genotype. Seven additional subjects provided written informed consent and were added to the data set, after which, it consisted of three cases in the following examination using the data of mucosal concentration of tacrolimus.

**Figure 5:**
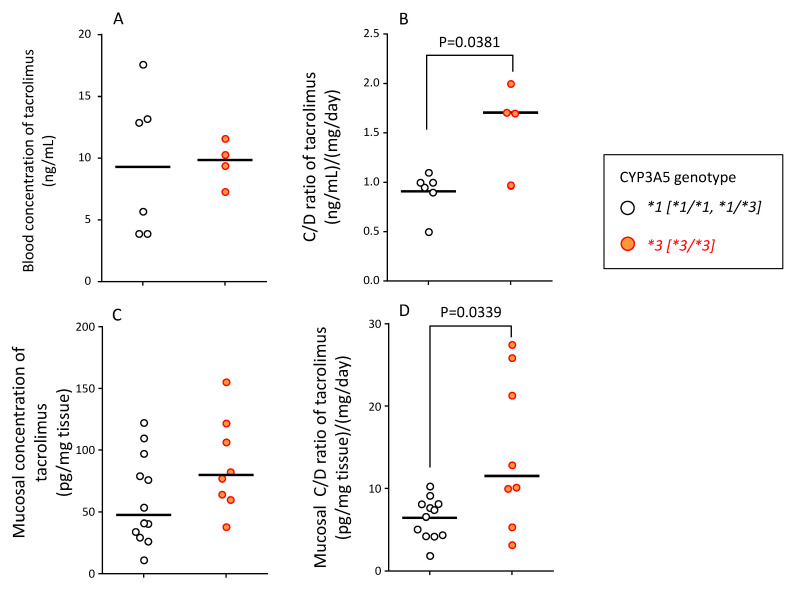
Relationship between *CYP3A5* genotype and blood concentration (**A**), blood C/D ratio (**B**), mucosal concentration (**C**), and mucosal C/D ratio (**D**) of tacrolimus at endoscopic examination. (**A**) The blood concentration of tacrolimus in patients carrying the CYP3A5*1 allele and CYP3A5*3/*3 genotype is shown. (**B**) The blood concentration/dose (C/D) ratio of tacrolimus in patients carrying CYP3A5*1 allele and CYP3A5*3/*3 genotype is shown. (**C**) The concentration of tacrolimus in the mucosal tissue of biopsy specimens at endoscopic examination is shown. Two biopsy specimens were taken per patient to measure the tissue concentration of tacrolimus to account for intra-individual variation. (**D**) The mucosal concentration/dose (C/D) ratio of tacrolimus in patients carrying the CYP3A5*1 allele and CYP3A5*3/*3 genotype is shown. Two data points per patient of C/D ratio were used to account for intra-individual variation. The bar shows the median in each group. Statistical analyses were performed using the Mann–Whitney’s *U*-test.

The tacrolimus blood concentrations (median (range)) in the CYP3A5*1 (active genotype, sum of *1/*1 and *1/*3, n = 6) and CYP3A5*3 (nonfunctional genotype, *3/*3, n = 4) were 9.3 (3.9–17.6) ng/mL and 9.9 (7.3–11.6) ng/mL, respectively (Figure 5A). The blood tacrolimus C/D ratio (median (range)) in patients carrying the CYP3A5*1 and CYP3A5*3 alleles was 0.96 (0.50–1.1) (ng/mL)/(mg/day) and 1.68 (0.98–2.04) (ng/mL)/(mg/day) (*p* = 0.0381), respectively (Figure 5B).

The mucosal concentration of unchanged tacrolimus (median (range)) in patients with CYP3A5*1 and CYP3A5*3 allele was 47.4 (11.3–122.4) and 79.9 (38.2–155.4) pg/mg tissue, respectively. Although there was no statistical significance, the mucosal concentration of tacrolimus was approximately 1.7-fold higher in patients with CYP3A5*3/*3 than in patients with CYP3A5*1/*1 and CYP3A5*1/*3 genotypes (Figure 5C).

The mucosal C/D ratio of tacrolimus (median (range)) in patients with CYP3A5*1 and CYP3A5*3 allele was 7.0 (1.9–10.3) (ng/mL)/(mg/mL) and 11.5 (3.2–27.5) (ng/mL)/(mg/mL), respectively, with a significant difference between these values (*p* = 0.0339) (Figure 5D).

## 3. Discussion

Tacrolimus and azathioprine, among other biological agents, are used in the remission induction of UC [28]. Among these, tacrolimus is one of the few oral therapeutic drugs that can be used in fulminant and steroid-refractory cases, sudden onset of disease conditions, or during deterioration of the existing condition, because a precise dose adjustment is easier for this drug than for others [3,29]. Therefore, the efficacy of tacrolimus in such conditions can be helpful in avoiding surgical treatment. However, a large inter-individual variation in its pharmacokinetics is closely associated with the frequency of its adverse reactions, which often leads to discontinuation of therapy [7,8]. In this study, we investigated the relationship between clinical efficacy of tacrolimus and the *CYP3A5* genotype in patients with UC.

The mRNA expression level of ABCB1 in the small-intestinal mucosa shows a negative correlation with C/D ratio of tacrolimus in liver-transplant patients [30]. In addition, downregulated Pgp and CYP3A contribute to cyclosporine blood level in the mice colitis model [31]. Since Pgp is located in the apical membrane and CYP3A5 is in the microsome, the decreased expression of Pgp may affect the absorption process of tacrolimus regarding the phenomenon in liver transplant patients [32]. Therefore, a decrease in mRNA expression level of ABCB1 in the inflammatory areas may influence tacrolimus pharmacokinetics via functional impairment of Pgp. Considering the good correlation between mRNA expression level and protein expression level in the intestinal plasma membrane fraction in the Japanese patients [32], the mRNA expression level of ABCB1 was determined to reflect the activity of Pgp as an efflux transporter in the colon epithelial cells. In the present study, the mRNA expression of ABCB1 (MDR1) was lower in inflamed areas than in the non-inflamed area in patients with UC (Figure 1C), comparable with the previous findings [26,27]. However, the inflamed regions of mucosa were limited in the relatively moderate patients and, therefore, the expressional change in MDR1 mRNA was suggested to influence not so much on tacrolimus pharmacokinetics in the present patients. Interestingly, the mRNA expression level of CYP3A5 was relatively maintained even in the inflamed mucosa (Figure 1B), suggesting mucosal CYP3A5 acts as detoxicating player regardless of inflammatory status in UC patients. Elucidation of difference in the molecular mechanisms of regulation in mRNA expression levels of MDR1 and CYP3A5 may be able to understand the maintained expression level of CYP3A5 in inflamed colon mucosa.

Our study revealed that the mRNA expression level of CYP3A5 was higher than that of CYP3A4 in non-inflamed colonic mucosa not only in healthy subjects, [33] but also in UC patients (Figure 1A). Furthermore, the C/D ratio of tacrolimus was about 2.5-fold higher in the CYP3A5*3/*3 genotype than in CYP3A5*1/*3 (Figure 2C) and was consistent with previous findings [24,34]. These results indicated that CYP3A5, rather than CYP3A4, was largely involved in the metabolism of tacrolimus in the colon, and the patients carrying CYP3A5*3/*3 showed a lower metabolism of tacrolimus compared to those carrying CYP3A5*1/*3. Because of maintained expression level of CYP3A5 mRNA in the inflamed mucosa compared to the non-inflamed mucosa (Figure 1B), colon CYP3A5 may actively metabolize drugs as well as tacrolimus regardless inflammation. In addition, attaining and maintaining blood-tacrolimus levels between 10 and 15 ng/mL at an early stage of administration is an important aspect in the treatment of UC [35]. These results put together suggested *CYP3A5* polymorphism as a useful biomarker in determining the initial dose and/or the dose escalation schedule of tacrolimus in the treatment of UC.

**Figure 6:**
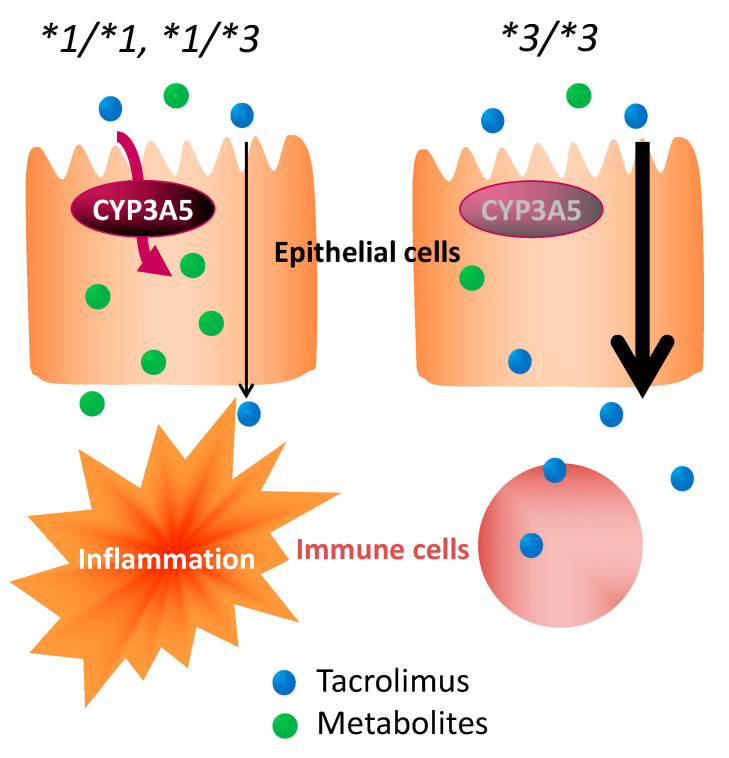
Impact of *CYP3A5* genotype in the pharmacological effect of tacrolimus in colon epithelial cells.

With no concomitant use of drugs potentially showing drug–drug interaction with tacrolimus in the present study, it was suggested that the effect of tacrolimus in the treatment of UC varied, depending on the polymorphism of *CYP3A5* (Figure 3 and Figure 4). These results suggested that the time to evaluate tacrolimus treatment for UC should be optimized based on the *CYP3A5* genotype. We hypothesized the following to explain these clinical results using molecular biology: The *1 or *3 genotypes reflect the activity or the nonfunctional of CYP3A5, respectively, which is a major enzyme of the CYP3A subfamily expressed in colon epithelial cells. Hence, orally administered tacrolimus would be extensively metabolized in the epithelial cells of the colon in patients carrying the CYP3A5*1 allele and, consequently, the immunocompetent cells would be deprived of the dose of tacrolimus required for optimal therapeutic effect (Figure 6). In contrast, a higher concentration of unmetabolized tacrolimus was found in the immune cells in the CYP3A5*3/*3 genotype. Therefore, the clinical evaluation of tacrolimus therapy in the management of UC requires more time in patients carrying the CYP3A5*1 allele than in those carrying the CYP3A5*3/*3. In fact, in the present study, there was an almost two-fold difference in the mucosal concentration of tacrolimus (Figure 5C) and its mucosal C/D ratio (Figure 5D) despite similar blood levels of tacrolimus among *CYP3A5* genotypes (Figure 5). Furthermore, the patients in the CYP3A5*1 group tended to be more frequently prescribed biological products after tacrolimus therapy during the 30 months’ survey after the start of tacrolimus therapy (3 out of 5 in CYP3A5*1/*1, *1/*3 versus 1 out of 6 in CYP3A5*3/*3; odds ratio (OR) = 7.5; 95% CI = 0.5–122.8). These results support our hypothesis (Figure 6). Tacrolimus is also metabolized in the liver, but tacrolimus has a gastrointestinal absorption rate of about 20%. Furthermore, the area under the concentration-time curve (AUC) coefficient of variation for oral administration and continuous IV injection is 72% and 42%, respectively. Therefore, it is considered that about 40% of individual variation in blood concentration after oral administration occur during the absorption process from the digestive tract. The cytochrome P450 (CYP) content of the digestive tract is considered as 1/80 compared to that in liver, but tacrolimus has a high protein-binding rate and, therefore, the metabolic contribution in the digestive tract cannot be important [7]. In liver-transplant patients, the *CYP3A5* genotype influences not only the tacrolimus-blood levels, but also the hepatic-drug levels, which may pose a potential risk of acute rejection after transplantation [36]. There are studies that report the efficacy of tacrolimus suppositories and enemas in UC [37]. Based on these backgrounds and present results, the *CYP3A5* polymorphisms were suggested to act as an important role in the local mucosal concentration of tacrolimus as well as systemic blood level of tacrolimus mainly affected by hepatic metabolism. Therefore, it was thought that not only the blood concentration but also the concentration at the inflammatory area is important for the therapeutic effect of tacrolimus against UC. Furthermore, *CYP3A4* and *CYP3A5* have haplotypes, but the most common CYP3A5*3/*3 is in combination with CYP3A4*1 (wild type) [38], and the CYP3A5*1 is in combination with CYP3A4*1G [39]. Considering almost no expression level of CYP3A4 in colon mucosa, it is unlikely that *CYP3A4* will affect the mucosal concentration of tacrolimus in patients with CYP3A5*3/*3. Further analysis may be needed to clarify the molecular mechanisms to affect mucosal concentration of tacrolimus in colon in addition to CYP3A5.

Based on these reports, the local concentration (mucosal concentration) was considered important for the therapeutic effect of tacrolimus. Our findings suggested that the local as well as systemic concentration of tacrolimus was important in the treatment of UC. *CYP3A5* polymorphism was closely associated with the mucosal concentration of tacrolimus (Figure 5). Therefore, the CYP3A5*3 genotype could be a useful biomarker in determining the optimal time to determine the clinical efficacy of tacrolimus in UC treatment.

In conclusion, it was suggested that the *CYP3A5* genotype not only affected the pharmacokinetics of tacrolimus, but is also associated with its efficacy in the management of UC. In addition, we found that the local concentration of tacrolimus may have played an important role in achieving optimal effects. These results indicated that the *CYP3A5* genotype could be a useful biomarker in tacrolimus therapy in association with treatment outcome with tacrolimus.

There were some limitations including the small sample size in this study. The data collection was limited in clinical situation and, therefore, all data sets were not collected from each patient, which is consistent of *CYP3A5* genotype, blood concentration of tacrolimus, mucosal concentration of tacrolimus, the change of CAI estimation and outcome such as cure, and surgery or prescription of biologics. In this study, we spent five years investigating four types of research subjects, but each research term was about 1 to 2 years, and there were not many patients who met each research condition. However, the ratio of *1/*1, */*3, and *3/*3 is about the same as the past reports targeting Japanese people [8,39,40]. In addition, the number of measurements of mucosal concentration of tacrolimus was limited in a couple of pieces per patients for ethical considerations. Therefore, further analysis with a larger sample size containing all data sets is required to assess the accuracy of the present results.

## 4. Materials and Methods

### 4.1. Materials

Analytical-grade tacrolimus was provided by Astellas Pharma Inc. (Tokyo, Japan). Sirolimus was purchased from LC Laboratories (Woburn, MA, USA) and used as an internal standard. The liquid chromatography-tandem mass spectrometry/mass spectrometry (LC-MS/MS) system was comprised of a high-performance liquid chromatography (HPLC) machine (LC-10AS; Shimadzu Corp., Kyoto, Japan), a column (Inertsil-ODS3, 150 mm × 2.1 mm i.d.; GL Sciences, Inc., Tokyo, Japan), and an MS/MS detector (API4000; Applied Biosystems, Foster City, CA, USA).

### 4.2. Subjects

**Figure 7:**
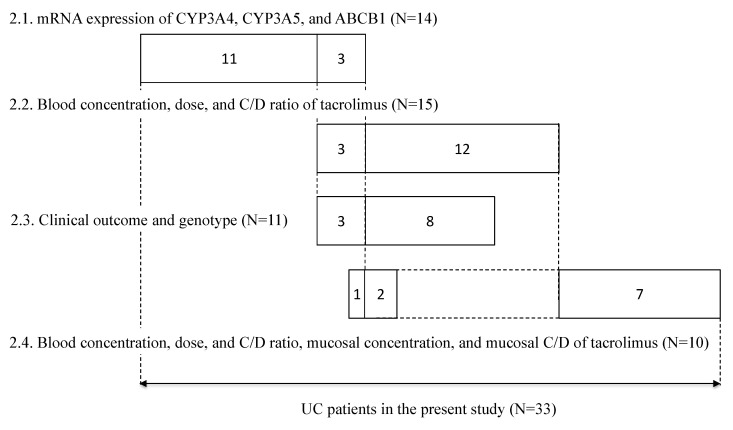
Summary of subjects enrolled in this study. The numbers in each box reflect the number of subjects. The numbers in each line correspond to each subsection of the results section, respectively.

Between May 2010 and May 2015, patients participating in the retrospective study at Kyoto University Hospital (Kyoto, Japan) provided written informed consent. Patients aged 15 years or younger and those treated with topical formulations were excluded from the study; a total of 33 patients were included in the study. Each patient underwent periodic sigmoidoscopy, in which mucosal biopsy samples (about 1-mm-cube each) were collected from a couple of areas in the rectum. The endoscopy was not done just for this study, but for taking biopsy specimens for pathological examination. The samples were put in a microtube, immediately frozen in liquid nitrogen, and stored at −80 °C until analysis to determine the mRNA expression levels of CYP3A4, CYP3A5, and ABCB1, and/or the concentration of tacrolimus. In 14 patients, the mRNA expression levels of CYP3A4 and CYP3A5 in non-inflamed colonic or rectal mucosa and the mRNA expression levels of ABCB1 in non-inflamed and inflamed colonic or rectal mucosa were examined. The relationship between tacrolimus concentration/dose (C/D) ratio and *CYP3A5* genotype in 15 patients treated with tacrolimus was examined. The relationship between the ratio of variation in CAI values and *CYP3A5* genotype was examined in 11 patients. The relationship of the mucosal concentration/dose ratio of tacrolimus and *CYP3A5* genotype was examined in 10 patients, on whom a biopsy was performed during tacrolimus therapy (Figure 7).

This study was conducted in accordance with the Declaration of Helsinki and its amendments, and was approved by the Kyoto University Graduate School and Faculty of Medicine, Ethics Committee (Approval number: G-408, 22 March 2011).

### 4.3. Measurement of mRNA Expression of CYP3A4, CYP3A5, and ABCB1

Mucosal biopsy specimens were homogenized in RLT buffer (QIAGEN, Hilden, Germany). Total RNA was isolated using MagNAPure LC RNA Isolation kit II (Roche, Mannheim, Germany) and reverse transcribed, as described previously [20]. The mRNA expression levels of ABCB1, CYP3A4, and CYP3A5 were evaluated by using real-time polymerase chain reaction (PCR) using an ABI prism 7700 sequence detector (Applied Biosystems, Foster, CA). The primer/probe sets used in this study were those reported by Koch et al. [41]. Glyceraldehyde-3-phosphate dehydrogenase (GAPDH) was used as an internal control, as described previously [42]. The mRNA expression data for each sample were corrected by the amount of GAPDH.

### 4.4. Isolation of Genomic DNA and Genotyping

Genomic DNA was isolated from a homogenate of mucosal biopsy specimens from patients, using MagNAPure LC DNA isolation kit I (Roche, Mannheim, Germany). The isolated DNA was used for genotyping using the PCR-restriction fragment length polymorphism method [41,42].

Genomic DNA was also isolated from the peripheral blood of patients, using EZ1 DNA Blood 350 µL kit (QIAGEN, Hilden, Germany). PCR and high-resolution melting (HRM) were consecutively performed using a LightCycler Nano (Roche, Mannheim, Germany). Probes and primers used for PCR were the same as those used for analysis of mucosal biopsy specimens. The PCR process included initial denaturation at 95 °C for 10 min, 45 cycles of denaturation at 95 °C for 10 s, annealing at 55 °C for 10 s, and synthesis at 72 °C for 15 s. The final HRM step was performed from 40 °C to 75 °C with an increase of 0.1 °C per second after denaturation at 95 °C for 30 s.

### 4.5. Measurement of Tacrolimus Blood Concentration

Routine analysis of the therapeutic drug monitoring of tacrolimus trough levels was conducted. Blood samples were collected before the morning dose into tubes containing ethylenediaminetetraacetic acid. Tacrolimus blood concentrations were determined using semiautomated chemiluminescent immunoassay (CLIA) according to the instruction manual (ARCHITECT, Abbott Japan Co., Ltd., Tokyo, Japan).

### 4.6. Measurement of Mucosal Concentration of Tacrolimus

Each patient was subjected to periodic sigmoidoscopy; mucosal biopsy tissues, each measuring about 1 mm^3^ were excised from one to three areas in the rectum. These samples were collected in a micro-tube, immediately frozen in liquid nitrogen, and stored at −80 °C until analysis. The individual weight of samples was measured. After each specimen was individually separated, it was transferred to a protein LoBind tube (Eppendorf Co, Ltd. Tokyo, Japan) and homogenized using ultrasonication in 300 µL of ultrapure water. The homogenates (300 µL) were transferred to glass tubes, spiked with 25 µL of the internal standard sirolimus (100 ng/mL), and vortexed for 10 s. Then, 600 µL of water and 2 mL of extraction solution (methyl-t-butyl ether or cyclohexane, 1:3 v/v) were added to the glass tubes. Each tube was capped securely, mixed on a horizontal shaker for 15 min, and centrifuged at 3000 rpm at room temperature for 10 min. The organic layer was transferred to another tube and evaporated using an Automatic Environmental Speed Vac^®^ System (Thermo Fisher Scientific Inc., Waltham, MA, USA) at 60 °C for 30 min. Each sample was reconstituted with 150 µL of the mobile phase (80% methanol with 1 mM ammonium acetate), vortexed for 1 min, and filtered using a 4 mm Cosmonice Filter W (Nacalai Tesque, Kyoto, Japan). LC-MS/MS protocols reported by Hosohata et al. were used [19].

### 4.7. Statistical Analyses

All statistical analyses were performed using Prism version 8 (GraphPad Software, Inc., San Diego, CA, USA). If the F distributions were the same, *t*-test was used. If they were different, nonparametric test was used. Mann–Whitney’s *U*-test and Kruskal–Wallis test were used to compare the differences between CYP3A5*1 (*1/*1 or *1/*3) and CYP3A5*3/*3 genotypes. A value of *p* < 0.05 was considered statistically significant.

## Figures and Tables

**Table 1:** Patient demographics for mRNA expression level measurement.

Patient	Number
Number	14
Age (years)	
Median (range)	41.5 (24–73)
Sex (male/female)	12/2
UC extent (number)	
Rectal	2
Left-side	3
Extended colon	9

**Table 2:** Patients characteristics based on *CYP3A5* genotype.

CYP3A5 Protein.	Expressor		Non Functional
*CYP3A5* genotype	*1/*1	*1/*3	*3/*3
Number	2	10	11
Age (years)			
Median (range)	35 (25–56)		35 (23–79)
Sex (male/female)	10/2		9/2
UC extent (number)			
Left-side	6		1
Extended colon	6		10

## Abbreviations

ABCB1	ATP binding cassette transporter B1
CYP	cytochrome p450
C/D	concentration/dose
UC	ulcerative colitis
FKBP12	FK506-binding protein 12
NFAT	nuclear factor of activated T
MDR1	Multidrug resistance 1
CAI	clinical activity index
TNF	tumor necrosis factor
LC-MS/MS	liquid chromatography-tandem mass spectrometry/mass spectrometry
HPLC	high-performance liquid chromatography
PCR	polymerase chain reaction
GAPDH	glyceraldehyde 3-phosphate dehydrogenase
HRM	high-resolution melting
CLIA	chemiluminescent immunoassay
ABCB1	ATP binding cassette transporter B1
